# Discriminating Intercalative Effects of Threading Intercalator Nogalamycin, from Classical Intercalator Daunomycin, Using Single Molecule Atomic Force Spectroscopy

**DOI:** 10.1371/journal.pone.0154666

**Published:** 2016-05-16

**Authors:** T. Banerjee, S. Banerjee, S. Sett, S. Ghosh, T. Rakshit, R. Mukhopadhyay

**Affiliations:** Department of Biological Chemistry, Indian Association for the Cultivation of Science, Kolkata, 700 032, India; LAAS-CNRS, FRANCE

## Abstract

DNA threading intercalators are a unique class of intercalating agents, albeit little biophysical information is available on their intercalative actions. Herein, the intercalative effects of nogalamycin, which is a naturally-occurring DNA threading intercalator, have been investigated by high-resolution atomic force microscopy (AFM) and spectroscopy (AFS). The results have been compared with those of the well-known chemotherapeutic drug daunomycin, which is a non-threading classical intercalator bearing structural similarity to nogalamycin. A comparative AFM assessment revealed a greater increase in DNA contour length over the entire incubation period of 48 h for nogalamycin treatment, whereas the contour length increase manifested faster in case of daunomycin. The elastic response of single DNA molecules to an externally applied force was investigated by the single molecule AFS approach. Characteristic mechanical fingerprints in the overstretching behaviour clearly distinguished the nogalamycin/daunomycin-treated dsDNA from untreated dsDNA—the former appearing less elastic than the latter, and the nogalamycin-treated DNA distinguished from the daunomycin-treated DNA—the classically intercalated dsDNA appearing the least elastic. A single molecule AFS-based discrimination of threading intercalation from the classical type is being reported for the first time.

## Introduction

In recent times, development of novel chemotherapeutic intercalators and understanding their actions, for treatment of malignancy, has become one of the primary research goals of medicinal chemists [1, 2]. Intercalation, defined as sliding-in of a planar aromatic ring system and its interaction with Π-electron system of contiguous DNA base pairs, can effectively lead to inhibition of DNA replication, invoking cytotoxicity. Amongst two major types of intercalators—threading and non-threading, threading intercalation is an unusual DNA binding mode where a chromophore (e.g., anthracycline) is inserted into the duplex and the terminal bulky groups are placed at the two dsDNA grooves [3], which is different from classical intercalation. Major biological implications of such concurrent barriers raised at both the grooves could be an enhanced ability of impeding DNA-protein interactions and effectively regulate gene expression [4], as well as slower dissociation from cellular DNA [4, 5]. An outcome of the difficulties in entry of the bulky side groups inside dsDNA structure, for intercalation to occur, could be manifested in significantly slower association rate as well [6].

**Fig 1:**
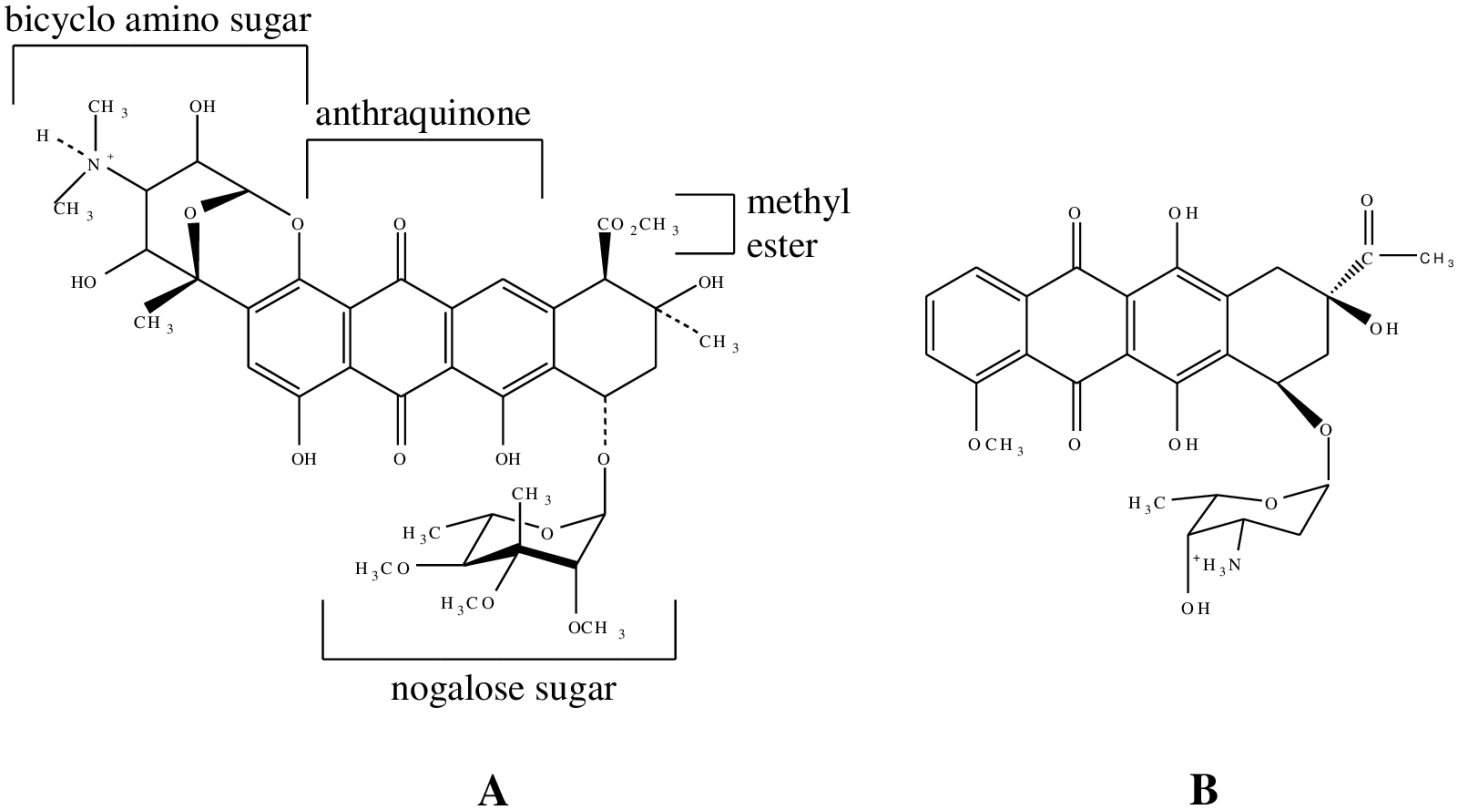
Structures of (A) nogalamycin and (B) daunomycin.

In this work, the intercalative actions of the naturally-occurring threading intercalator nogalamycin (from *streptomyces nogalater* bacteria), which is an antibiotic [7], antitumour/anticancer [8, 9] agent, have been investigated. In order to draw comparison to classical intercalation and identify the unique effects of threading intercalation, the antibiotic antitumor drug daunomycin [10] (from fungus belonging to *streptomyces* family) having structural similarity to nogalamycin [Fig 1], has been studied too. In both cases, DNA binding is known to cause DNA structural alterations that interfere with enzymatic actions (e.g., inhibition of topoisomerase II), which are essential for DNA replication and transcription, eventually leading to cell death, since the crucial processes like DNA synthesis and repair, RNA synthesis and transcription, are compromised with [8,11,12].

So far, X-ray crystallographic [13, 14] and NMR [15] studies have been performed to obtain quantitative information on nogalamycin-treated DNA, although on short hexameric sequences. Herein, high-resolution AFM, an established method for single molecule detection [16–18], has been applied to obtain direct visual information on intercalator-induced DNA structural changes, using longer sequence (692 bp) than those previously studied [13–15]. The alterations in dsDNA mechanical properties, upon drug treatment, have been monitored by recording force-extension profiles of the stretched DNA molecules by single molecule atomic force spectroscopy (AFS). This approach is well-known for obtaining detail information on the forces operating behind structural organization of biopolymers and changes in it due to interactions with small molecules with pN (10^−12^ N) level force sensitivity [19, 20, 21, 22] and proteins [23].

In a previous study on intercalative effects of nogalamycin, a gradual change from predominantly relaxed form having irregular supercoiling to the more compact plectonemic superhelix was observed [24]. However, a comparative treatment of the effects of threading and non-threading intercalation has not been reported. Herein, we present a quantitative account of the effects of both the drugs on dsDNA molecules. While clear indications of intercalative drug action could be obtained from molecular lengthening upon drug treatment [25], and from the structural transition (B-form to stretched S from, in which most of the DNA base stacking is lost [26]), for both the drugs, certain differences could be discerned, which will be presented in this report.

## Materials and Methods

### Preparation of DNA/DNA-drug solutions

Preparation of dsDNA fragments: See S1 File.

Preparation of DNA-Nogalamycin complex: Nogalamycin (Sigma) was dissolved in 20 mM HCl (pH adjusted to 7.0) and diluted with TE buffer (pH 7.0) as required, prior to preparation of the DNA-drug complex. The complex was prepared using low drug amounts, at DNA:drug molar ratios 1:10, 1:26 and 1:40. The intermediate value 1:26 was chosen considering -tgc- and -gca- as the primary binding sites, since these sites are thought to be the highest binding affinity sites for nogalamycin [15]. In the present sequence, -tgc- and -gca- appear 26 times. Drug-treated DNA solution was incubated at 37°C in dark for 1/6/12/18/36/48 h prior to adsorption onto APmica.

Preparation of DNA-Daunomycin complex: Daunomycin hydrochloride (Sigma) was dissolved in water and concentration determined using ε 11,500 M^-1^ cm^-1^ at 480 nm [27]. DNA-daunomycin complex was prepared under similar conditions as in case of nogalamycin, for time periods 1/18/36/48 h prior to adsorption onto APmica.

The vials containing drug solutions/DNA-drug complexes were kept well-covered with Al-foil to prevent DNA photodegradation in all cases.

### Sample preparation for AFM/AFS experiments

Preparation of APTES treated mica (APmica): As per reported protocol [24].

Preparation of sample with free DNA: See S1 File.

Preparation of sample with DNA-drug complex: Sample preparation method was kept same as that for free DNA, for both imaging and force spectroscopy experiments.

Preparation of sample for control experiments: See S1 File.

### Data acquisition and analysis

AFM/AFS experiments: AFM experiments were performed using PicoLE AFM (Agilent Corp., USA) and imaging carried out in ambient using cantilever (μmasch, Estonia) of frequency ~330 kHz and force constant 27.5 N/m in the intermittent contact mode. Amplitude set point was 85–90% of free oscillation (8.0 V) and scan speed 1.0–1.5 lines/s. All data presented were verified by sampling at least three different areas of the sample. Data processing was carried out using an in-built image processing software, where processing was limited to leveling via plane correction only. The DNA contour length was measured by tracing each DNA molecule with segmented lines. Then the length of each segmented lines was summed up to get the contour length in nm. The contour length measured this way was further verified by Image J software using the “segmented line” tool [28]. In general, the distances between the two adjacent points placed on the DNA contour to trace the molecule were few nm apart. These point-to-point distances were much less than the persistence length value of dsDNA, which is 50 nm [29, 30]. Hence, it provides considerably reliable contour length values. However, choosing a specific model for tracing the DNA contour is important, particularly if DNA molecule is long (few μm in length) and undergoes bends at a number of places. In those cases where the DNA bending is probed from the AFM topographs, simple WLC model may not be able to represent the DNA behavior at multiple length scales [31]. The width and height values were determined at three equidistant points along the molecular long axis, followed by averaging. Width values were measured as full width at half maximum of cross-sectional diagram, and height values measured as the difference between the highest point in cross-sectional diagram and average baseline. Only those molecules that were fully imaged within the selected scan area, without having any overlap with other molecules, were considered for quantitative analysis. Dimensional analyses were performed on 30–40 molecules each. All the deviations from mean as shown are standard deviations.

For force spectroscopy, cantilevers (Si_3_N_4_-Microlever, Bruker) of spring constants 24–25 pN.nm^-1^ (calibrated by thermal fluctuation method [19]) were used. The dsDNA strands, adsorbed onto gold surface, were mechanically contacted with tip by applying 1nN contact force and extended with 1 μm.s^-1^ piezo velocity. All the measurements were performed in identical TE buffer solutions at room temperature. The displayed force curves consist of about 9000 data points. The force-distance plots were corrected for cantilever bending by determining tip curvature under a certain force—the cantilever sensitivity S, and finding the exact distance (Z) between sample and tip, given by Z = L—(A/S), where L is the distance travelled by the piezo and A is the deflection signal in Volts [32]. Plots were smoothened by a box smoothing program window size 8 points using OriginPro 8 software [19]. All the force curves presented here are representative of the most frequently observed behaviour.

## Results

In this work, the unique intercalative effects of nogalamycin, which is taken as a representative of threading intercalators and an experimental model system, have been probed, in comparison to daunomycin, a non-threading intercalator, by high-resolution AFM imaging and single molecule AFS. All the samples were studied onto APmica, as negatively charged DNA can be electrostatically anchored onto positively charged 3-aminopropyltriethoxysilane (APTES), for effective DNA immobilization—a prerequisite for successful high-resolution AFM imaging. Onto APmica, the DNA molecules get kinetically trapped [29], and consequently, one can observe the solution conformations of DNA molecules, projected in 2-dimensions [33]. APmica serves to be a proper choice when the purpose is not to study a dynamic process, but to study the adsorbed static DNA molecules/DNA-drug complexes closely representing their respective solution conformations [34]. For acquiring force-extension data using single molecule force spectroscopy, dsDNA molecules were placed onto gold substrate and subsequently stretched by applying pN order forces [19].

**Fig 2:**
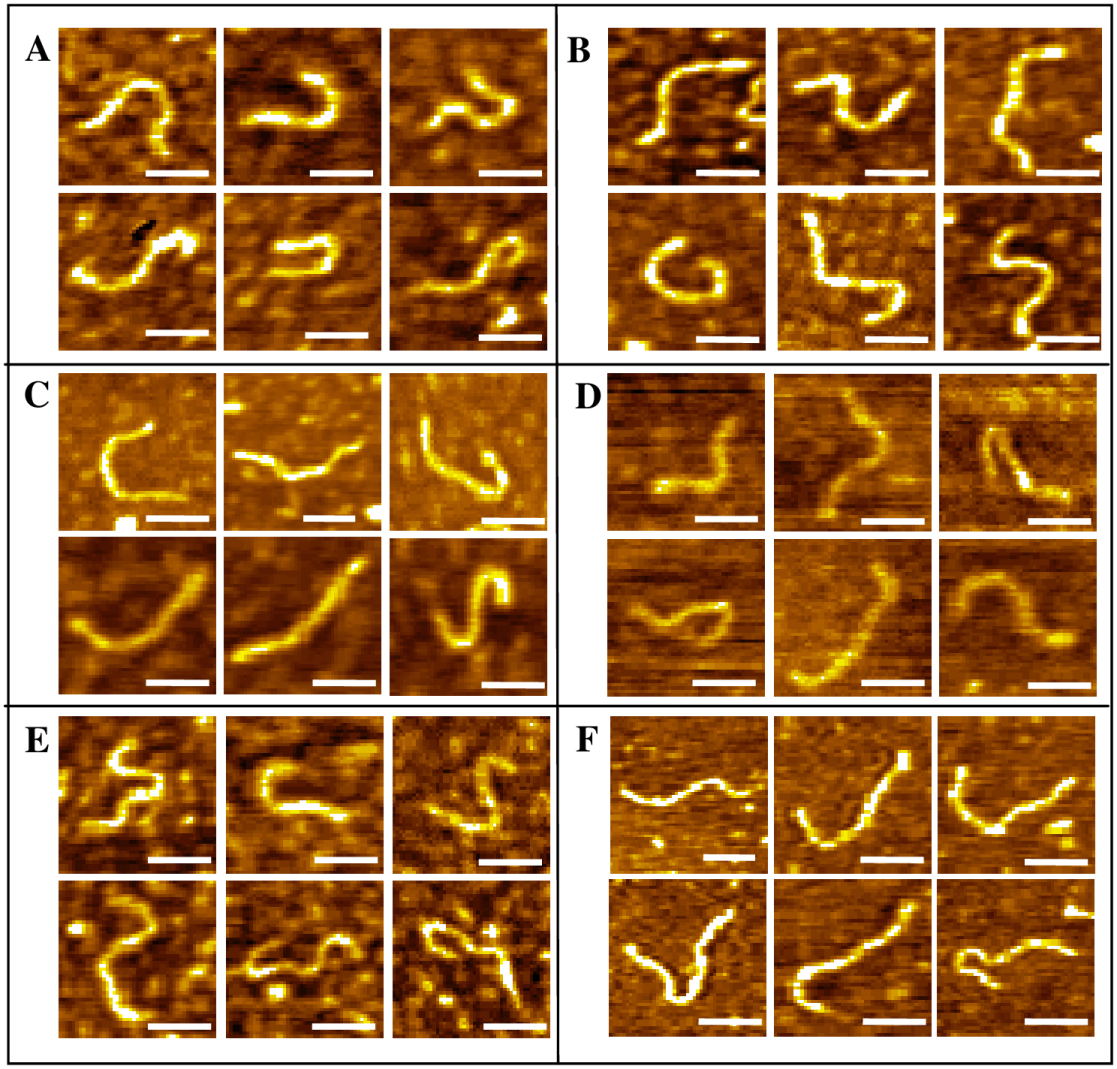
AFM topographs of DNA-nogalamycin complex after incubation for (A) 1 h, (B) 6 h, (C) 12 h, (D) 18 h, (E) 36 h and (F) 48 h. DNA_conc_: 1.7 μg.ml^-1^. Scale bar: 100 nm. Z-ranges: (A) 0–0.7 nm, (B) 0–0.8 nm, (C) 0–1.4 nm, (D) 0–0.6 nm, (E) 0–0.8 nm and (F) 0–0.8 nm.

**Fig 3:**
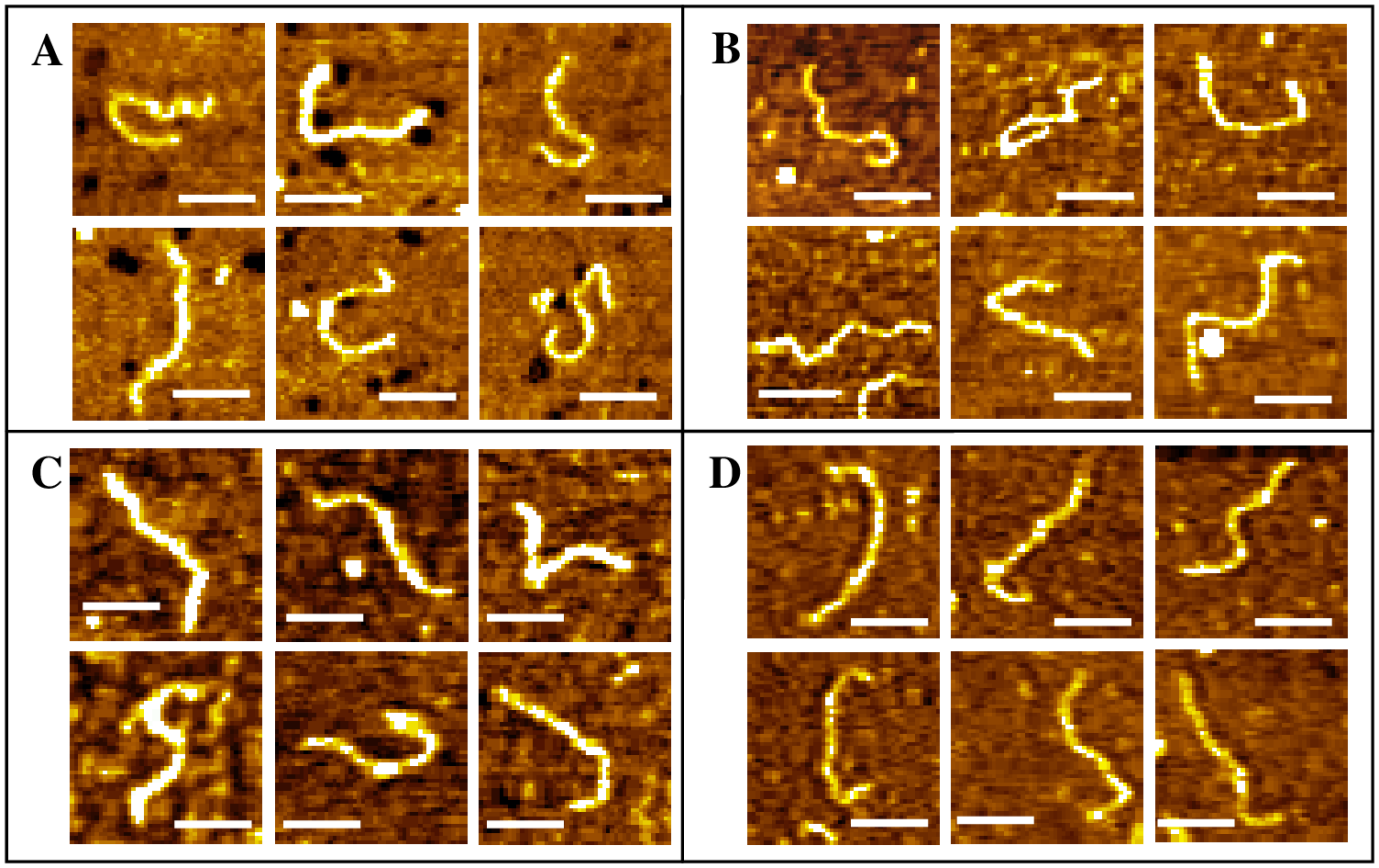
AFM topographs of DNA-daunomycin complex after incubation for (A) 1 h, (B) 18 h, (C) 36 h and (D) 48 h. DNA_conc_: 1.7 μg.ml^-1^. Scale bar: 100 nm. Z-ranges: (A) 0–1.2 nm, (B) 0–0.9 nm, (C) 0–0.8 nm and (D) 0–1.2 nm.

A number of conformations were observed for untreated dsDNA [Figure A in S1 File], and their average contour length was estimated as 217.0±25.7 nm, having a modest agreement with the theoretical length of 235.28 nm for 692 bp dsDNA (assuming B-form of DNA and 0.34 nm/bp rise), indicating B-form being mostly preserved on surface. The average width values of the fragments were estimated to be 14.4±3.08 nm, which is substantially broadened compared to the crystallographic width 2 nm—a typical AFM observation associated to tip-sample convolution [35]. AFM topographs of the nogalamycin-treated fragments indicate almost no change in the molecular shapes, even over the total incubation period [Fig 2], although a gradual increase in DNA contour length with increasing incubation period was revealed [Table A in S1 File]. No drastic change in the shapes of the daunomycin-treated DNA molecules could be observed either [Fig 3], although the contour length increased with increasing incubation time period [Table B in S1 File]. The DNA contour length, in case of nogalamycin-treated DNA, increased nearly 39.3% of the total increase (over 48 h incubation) within the first 1 h incubation [Table A in S1 File], whereas, about 86.2% of the total increase (over 48 h) took place within the first 1 h incubation in case of daunomycin treatment [Table B in S1 File]. Clear DNA contours could be observed even after continuous scanning (4–5 cycles) over the same area, indicating stable imaging during the experiments. The control experiments performed with the drug solution and TE buffer revealed only particulate features (not shown). Interference to intercalative binding due to covalent adduct formation with a photooxidized form of the drug molecules can be ruled out here, since the drug solutions were freshly prepared and maintained in dark (vials kept well-covered with Al-foil). From the UV-visible spectra of the drug solutions, stable absorbance peak near 490 nm (due to anthraquinone) was observed in accordance to earlier reports [36, 37].

**Fig 4:**
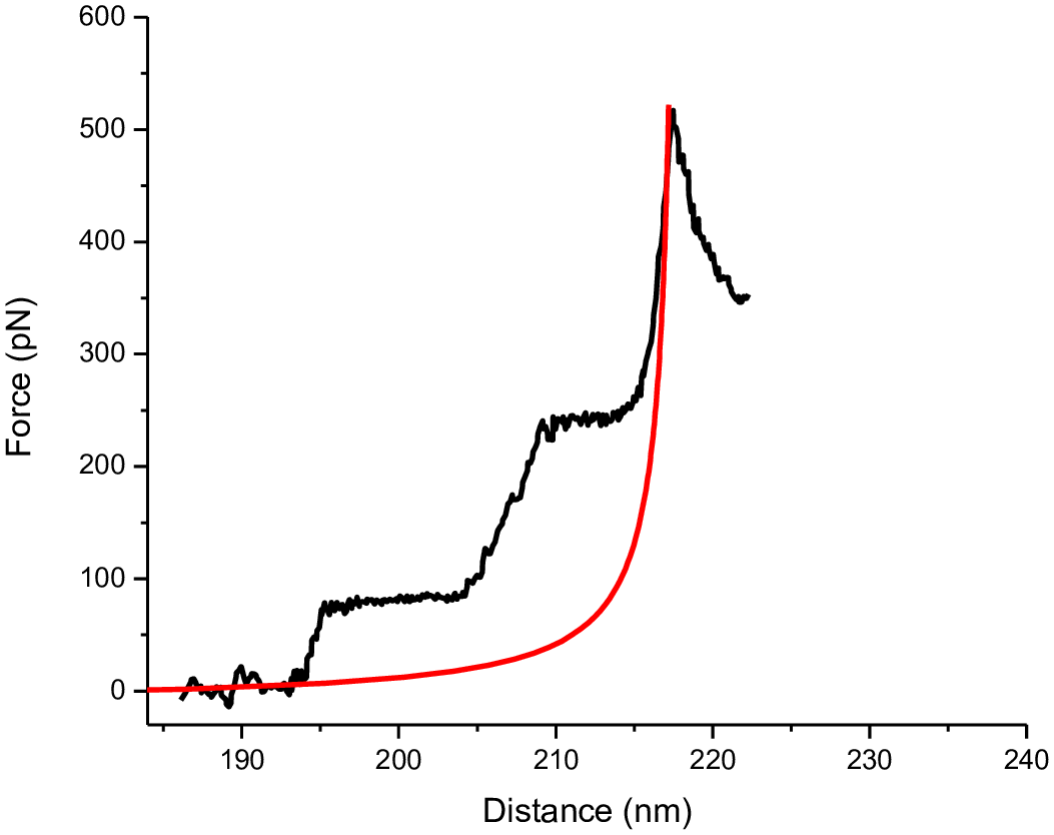
AFM force-extension trace for 692 bp dsDNA molecule. The red curve is the elasticity fit (well-fit to the high force ssDNA elasticity region) according to the modified FJC model having contour length (L) = 218 nm, Kuhn segment length (b) = 0.52 nm, molecular stretch modulus (S) = 800 pN at T = 25.4°C.

**Fig 5:**
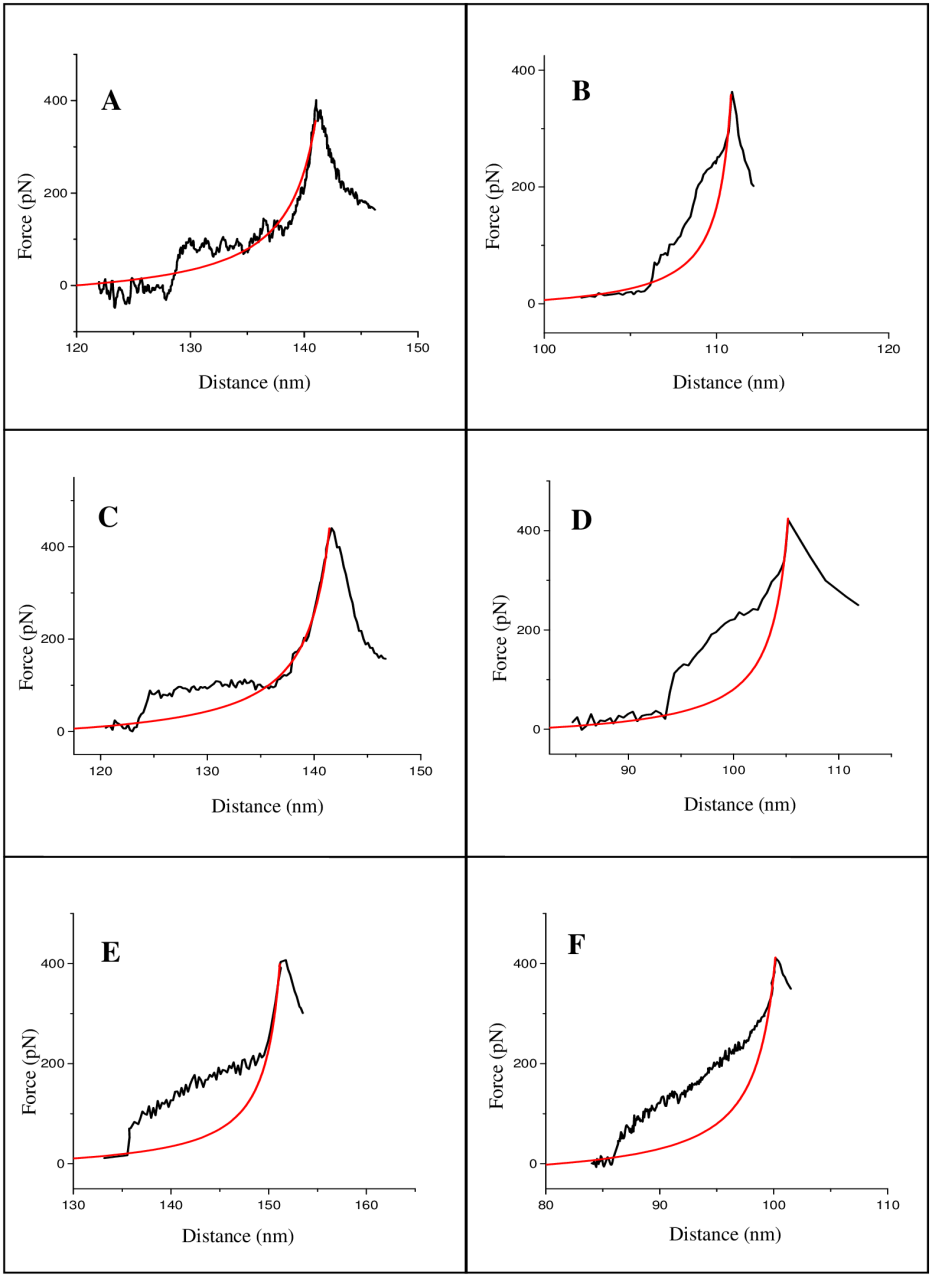
AFM force-extension traces for nogalamycin-treated dsDNA molecules, for dsDNA:drug molar ratio (1:10) after (A) 1 h and (B) 48 h incubation; for the molar ratio (1:26) after (C) 1 h and (D) 48 h incubation; and for the molar ratio 1:40 after (E) 1 h and (F) 48 h incubation. The red curves are the elasticity fits (well-fit to the high force ssDNA elasticity regions) according to the modified FJC model.

**Fig 6:**
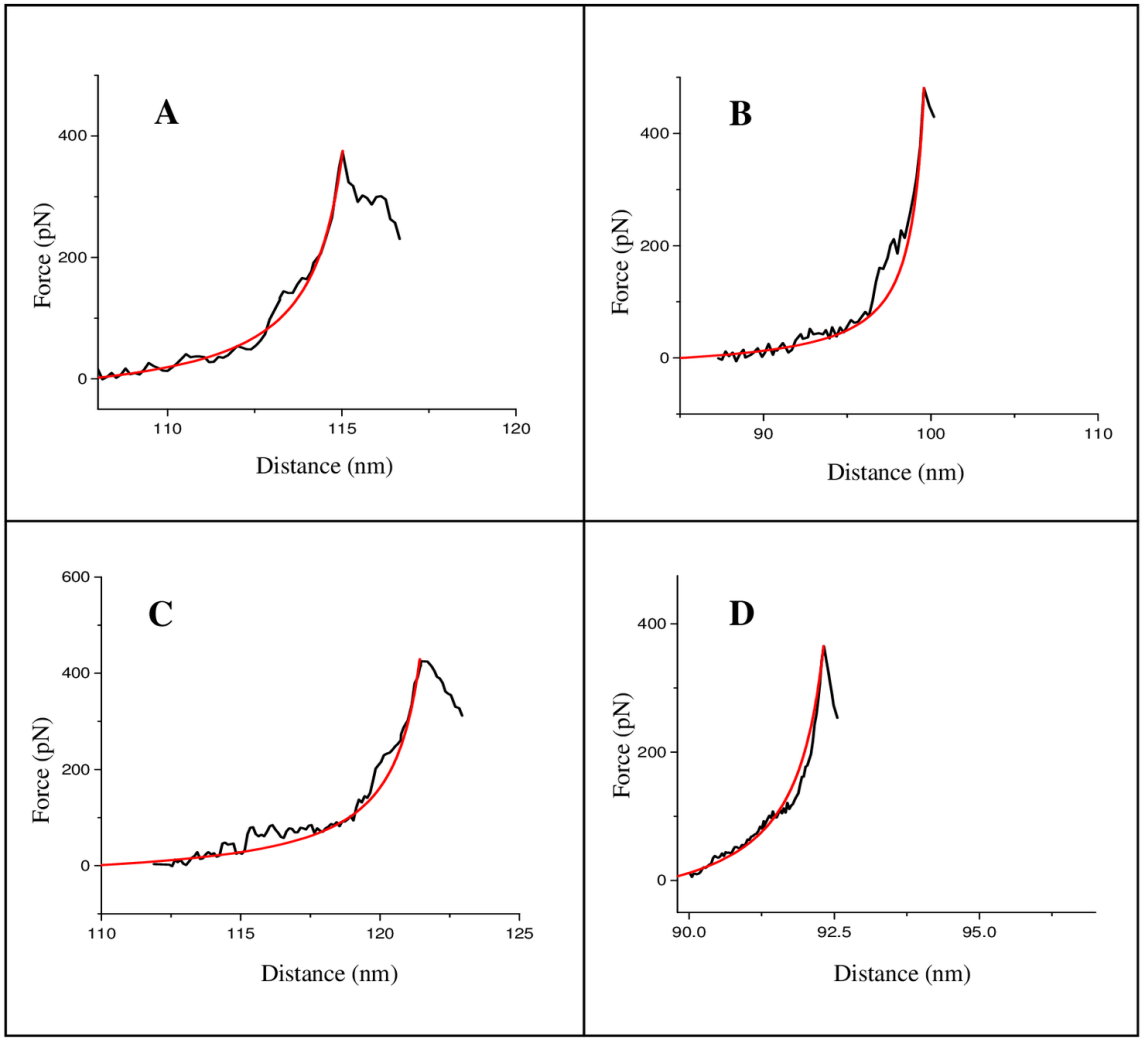
AFM force-extension traces for daunomycin-treated dsDNA molecules, for the dsDNA:drug molar ratio (1:10) after (A) 1 h and (B) 48 h incubation; and for the molar ratio (1:26) after (C) 1 h and (D) 48 h incubation. The red curves are the elasticity fits (well-fit to the high force ssDNA elasticity regions) according to the modified FJC model.

In Fig 4, the representative AFM force-extension profile of a single untreated dsDNA molecule, in which distinct signatures of intramolecular structural transitions [26, 38] can be detected, is shown. A plateau indicating co-operative transition from B-form to the overstretched S-form of dsDNA, where both 'B' and 'S' conformation can co-exist, was observed at 75 pN (±1.4 pN). It is generally understood that during B-S transition, majority of the base pair stacking interactions are lost, and 'S' DNA is formed, without rupture of the inter-strand hydrogen bonds, and therefore, this transition does not reflect melting to ssDNA [26]. Further, a mechanically induced melting transition from dsDNA to ssDNA was detected at 250 pN (±6.9 pN), and the elasticity fit to the ssDNA elasticity region, according to the modified freely jointed chain (FJC) model [39] (see S1 File for a description of the model) that depicts force-extension profile of a long, single polymeric molecule under stretched condition, revealed a contour length value (L) of 218 nm (± 0.48 nm). It is to be noted that this model is only applicable to fit properly the melting region, and not the full regime of the force-extension profile. In Fig 4, modified FJC model perfectly fits the melting region (beyond the force of ~250 pN) and this is applicable for all other force-extension profiles shown [Figs 5 and 6]. In addition, the parameters like Kuhn segment length (b) and molecular stretch modulus (S) (or molecular spring constant per unit length), as extracted from the fit along the ssDNA elasticity region, were found to be 0.5 nm and 800 pN, respectively, in close agreement with the previous reports [19, 38, 39].

The DNA force-extension profiles clearly changed after drug treatment (representative curves shown in Figs 5 and 6), as the corresponding molecular mechanics of the biopolymer altered upon drug-binding. In case of nogalamycin treatment, although overstretching transition could be detected in the initial phase of treatment [Fig 5A, 5C and 5E], cooperativity was reduced for the highest molar ratio applied [Fig 5E]. The cooperative nature of the overstretching transition was all together absent when drug treatment prolonged till 48 h [Fig 5B, 5D and 5F]. Furthermore, the melting transition to ssDNA, as observed in case of free dsDNA at 250 pN [Fig 4], could not be clearly detected [Fig 5A–5F]. Instead, a sharply rising ssDNA elasticity region that fit well with the FJC model, and was often preceded by a small hump finishing at 270–300 pN [Fig 5B, 5D and 5F], could be observed. The absence of a distinct melting transition could be related to intercalation-related DNA unwinding [40] and resultant weakening of the inter-strand H-bond interactions. It has been reported earlier that in case of poly(dA-dT), no melting transition can be detected, whereas in case of poly(dG-dC), distinct melting transition can be distinguished [38], meaning that weaker H-bond interactions are reflected in disappearance of the melting transition plateau. However, the higher forces required to reach the ssDNA elasticity region [Fig 5B, 5D and 5F] could be due to formation of a more stable and unique intercalated structure due to threading type of intercalation.

In case of daunomycin treatment, a faster and more linear increase of the force response at smaller extensions was observed (representative curves shown in Fig 6), compared to nogalamycin treatment [Fig 5]. The final peak occurring at shorter distances probably mean a less flexible biopolymer being generated in case of daunomycin treatment. Since the AFM tip can pick up a DNA molecule at any arbitrary point along its contour, which is reflected in varying length (designated as distance in X axis) from molecule to molecule [41], the observation of the final peak at shorter distances cannot be convincingly concluded to be due to a decrease in the length of the drug-treated DNA molecules. Our assumption that the DNA molecules became less flexible upon drug treatment can be supported by the end-to-end distance values, since the end-to-end distance value for the daunomycin-treated DNA molecules was found to be 248.5±19.2 nm (after 48 h treatment), whereas that for free DNA was 138.5±17.1 nm. Such a clear increase in the end-to-end distance value, indicating reduced flexibility of the DNA molecule upon drug treatment, was observed for the nogalamycin-treated DNA too, as the end-to-end distance value in this case was found to be 241.8±12.2 nm after 48 h treatment. No clear overstretching transition plateau could be detected in either of the four cases studied [Fig 6A–6D], meaning a less co-operative structural transition occurring. A minor slope, compared to the free dsDNA melting transition, starting at around 80 pN, was observed in all cases [Fig 6A–6D]—such absence of clear melting could mean that daunomycin-treated DNA was more rigid compared to untreated DNA. The ssDNA elasticity region in most cases was preceded by a small hump finishing at 130–170 pN, closely similar to those reported earlier [41].

## Discussion

In this study, a non-linear force response that is typical of a chain-like biopolymer [19] could be observed reproducibly, for both untreated [Fig 4] and intercalated dsDNA molecules [Figs 5 and 6] (see Figures D-F in S1 File for a couple of curves for each situation studied). We could distinguish a successful stretching event in about 60% cases, where the characteristic retrace profiles as shown in Figs 4, 5 and 6 were obtained. Since the intramolecular structural transitions, as observed for free dsDNA, can be attributed to the distinct topology and the base pair stacking patterns, any change(s) in the transitions in case of the intercalated DNA complexes can be ascribed to a change in the structural characteristics of dsDNA. The specific differences in the dsDNA force-extension profiles, for nogalamycin-treated and daunomycin-treated DNA, as delineated below, could then be attributed to dissimilarities introduced in dsDNA structure upon drug treatment.

### I. B-S overstretching transition is detectable solely in case of nogalamycin

According to a theoretical study, cooperativity in overstretching transition can be strongly dependent on base stacking in DNA double helix [42]. It is therefore possible that different perturbations of the stacking interactions would result into distinctive force curve profiles. No clear detection of B-S overstretching plateau in case of daunomycin-treated dsDNA [Fig 6], within the force pulling time scale, indicates rapid association of drug molecules, and occurrence of fast non-cooperative DNA structural changes. In case of nogalamycin, the observation of overstretching plateau in the first 1 h of treatment [Fig 5A and 5C] indicates cooperative DNA structural changes during the commencing phases of drug treatment. However, upon prolonged exposure to nogalamycin, loss of cooperativity becomes apparent and the transition cannot be distinguished from melting transition [Fig 5B, 5D and 5F]. The observation of the overstretching plateau in case of nogalamycin treatment [Fig 5A and 5C] may indicate a more elastic structure to be prevailing initially, as a result of slower intercalation by nogalamycin, compared to daunomycin. It is likely that at this stage, where the drug molecule has been attached to dsDNA although intercalation is yet to set in, nogalamycin acts as a groove-binder. This is possible, especially since nogalamycin possesses a positively charged side group (bicyclo amino sugar containing a tertiary ammonium) that may engage itself in electrostatic interactions with the negatively charged DNA backbone. It was shown earlier that intramolecular B-S transition remains visible as a distinct plateau in the force-extension trace in case of the groove-binding ligands [41], albeit at a lower force regime than in case of free dsDNA, whereas the plateau vanishes in case of intercalation [26, 41]. Pure groove-binding behaviour of nogalamycin in the initial 1h time can however be ruled out, since contour length value increased within this time period [Table A in S1 File], which is indicative of intercalation.

### II. Melting transition for nogalamycin occurs at a higher force regime than for daunomycin

The force-extension profiles revealed that nogalamycin-treated DNA molecules offered greater resistance to force-induced melting transition (at 270–300 pN) of dsDNA molecules to the ssDNA form [Fig 5], compared to daunomycin-treated DNA (at 130–170 pN) [Fig 6]. This means greater stability of the nogalamycin-treated DNA, probably as a result of a distinctive intercalated structure, where the two bulky side groups reside in the minor and major grooves, due to threading type of intercalation [43, 44], exerting a 'clipping or fastening' effect.

### III. Nogalamycin binds to DNA slower than does daunomycin

**Fig 7:**
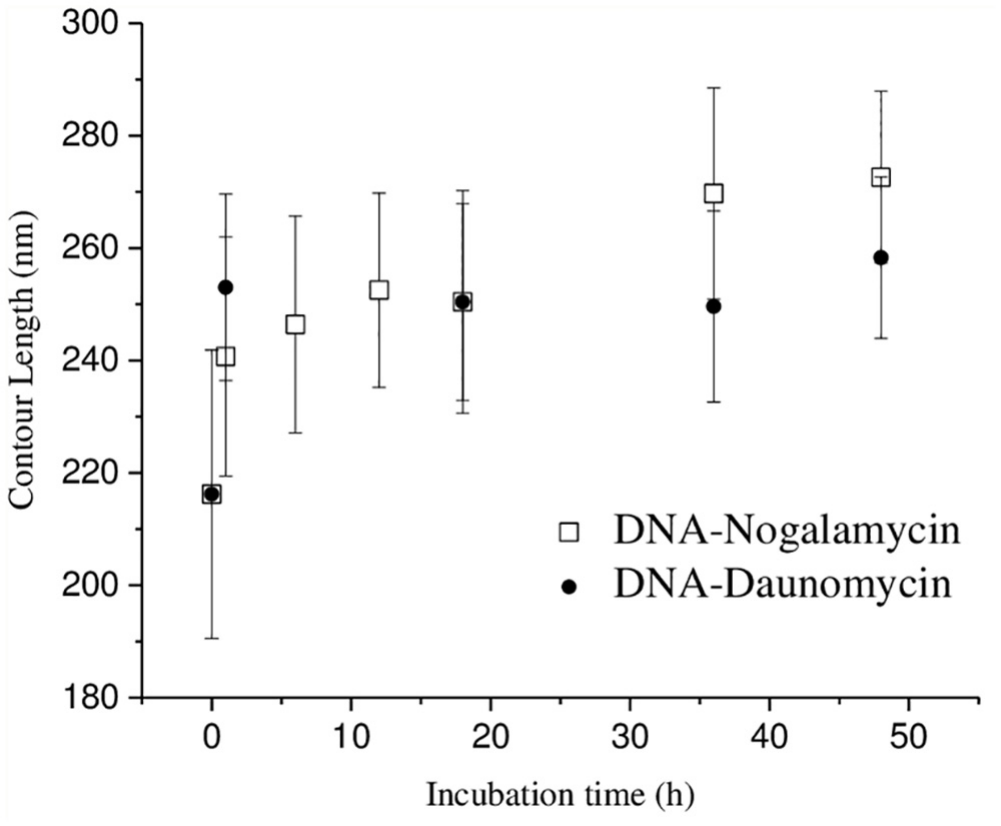
Changes in contour length of 692 bp dsDNA incubated with nogalamycin (open square) and daunomycin (filled circle) with time.

In the first 1 h of drug treatment, a drastic increase (86.2% of the total increase) in the contour length of daunomycin-treated DNA [Table B S1 File], in agreement with rapid structural changes as depicted in an AFS [33] and an optical tweezer [34] study, and only 39.3% of the total increase in case of nogalamycin-treated DNA [Table A S1 File], indicate slower incremental binding by the threading intercalator [Fig 7]. Earlier ensemble studies on nogalamycin indicated slow intercalation too that could involve few hours up to few days of treatment [13, 45, 46], as a result of a sterically-challenged process since the minimum width of nogalamycin molecule is about 1.2 nm while the DNA intercalation site can be opened till only 1.0 nm, only when the intermolecular hydrogen bonds (between the base pairs) were rapidly cleaved, making entry of the drug molecule possible, and then reformed, thus regenerating the base pairs and completing the process of threading intercalation [25, 26].

Although no clear information on sequence-selectivity of the two drugs is obtainable from this study, the observation that nogalamycin exhibits incremental monotonic increase in the contour length, whereas daunomycin exhibits a drastic increase in the initial phase, followed by small changes in the subsequent stages [Fig 7], could be due to differences in the sequence-recognition pattern of the two drugs. It has been suggested that nogalamycin binds best to those regions of DNA that are most easily disrupted yet that can be strongly stabilized by the presence of the drug [47]. Due to its unique structure, nogalamycin is also expected to show a restricted ability to interact with many types of DNA sequence stretches. Such restricted association, compounded with a low dissociation rate in case of nogalamycin binding, could lead to the observed incremental increase in contour length. A small dissociation rate constant (*k*_d_ = 0.001 s^-1^) was determined for nogalamycin in complexation with calf thymus DNA, which is in sharp contrast to the classical intercalators, such as ethidium bromide, which has a rate constant *k*_d_ = 13.4 s^-1^ [48,49]. In case of daunomycin, the dissociation rate constant value of 3.3–4.8 s^-1^, indicative of low affinity binding, was reported [50]. By comparison to nogalamycin, daunomycin apparently exhibits lesser selectivity, since it binds to every possible binding site, leading to rapid contour length increase in the initial 1 h drug treatment. Subsequently, some redistribution of the drug molecules could take place unlike in case of nogalamycin treatment, because rate of dissociation of daunomycin is higher compared to nogalamycin, causing some fluctuations in the contour length, although within a small range.

## Conclusions

In this work, AFM-based single molecule force spectroscopy has been applied to access the mechanical identity of dsDNA molecules, before and after treatment with two different types of intercalators—threading and non-threading/classical intercalator. Using subnanometer spatial resolution and piconewton force sensitivity offered by AFS, the detail changes in dsDNA molecular mechanics due to intercalator binding have been detected. Distinct differences in the contour length values and the force curve profiles of nogalamycin-DNA and daunomycin-DNA complexes, could be identified by AFS within the first 1 h of drug treatment. This is biologically relevant, since it has been shown that daunomycin treatment for 1 h is drastic enough to reduce the mitotic index of cells, meaning that the process of mitotic blockage initiates in the first 1 h of drug treatment [51]. Although the DNA structural changes upon nogalamycin treatment were observed to set in slower than in case of daunomycin treatment, the former case could mean more effective DNA damage being settled, as the nogalamycin-DNA complex was found to be more stable, as it required higher forces for melting, than the daunomycin-DNA complex.

## Supporting Information

S1 File Preparative and AFM data analysis related details. (DOC)
