# Supplementary material for: Discriminating Intercalative Effects of Threading Intercalator Nogalamycin, from Classical Intercalator Daunomycin, Using Single Molecule Atomic Force Spectroscopy
Source: PLoS One. 2016 May 16;11(5):e0154666. doi: 10.1371/journal.pone.0154666 (PMC4868319; doi:10.1371/journal.pone.0154666)
Supplement: S1 File — (DOC) [file pone.0154666.s001.doc]

**Supporting Information**

**Discriminating Intercalative Effects of Threading IntercalatorNogalamycin, from Classical IntercalatorDaunomycin, using Single Molecule**

**Atomic Force Spectroscopy**

T. Banerjee, S. Banerjee, S. Sett, S. Ghosh, T. Rakshitand R. Mukhopadhyay*

*Department of Biological Chemistry, Indian Association for the Cultivation of Science,*

*Kolkata-700 032, India*

###### Preparation of dsDNA fragments: The 692 bpdsDNA fragments were prepared by restriction digestion of pBR322 plasmid (Bangalore Genei, India) with *Dra*I (Bangalore Genei, India) enzyme, separated by gel electrophoresis on 0.7% low-melting agarose (Bangalore Genei, India) gel at r.t. (24±1 °C) in 0.5X TBE (Tris-borate/EDTA) (Bangalore Genei, India) buffer at 30 V/cm for 3 h, and extracted using a gel purification kit (Qiagen) in TE buffer (10 mMTris-HCl, 1 mM EDTA, pH 7.4). Unless mentioned, all sample solutions were prepared with autoclaved filtered Milli-Q water (resistivity: 18.2 MΩ.cm).

Preparation of sample with free DNA: For imaging experiments, 10µl DNA solution (conc. ~1.7 μgml^-1^) was deposited onto APmica, kept for 20 min in ambient (r. t., humidity ~40%), followed by gentle washing with 2 ml TE buffer (pH 7.4) and 5 ml water, and drying with a gentle stream of nitrogen gas. For force spectroscopy, 1.0 μgμl^-1^ DNA solutionwas deposited onto freshly annealed gold(111) surface, dried at ambient conditions, sample surface gently washed with 2 ml (8×250µl) 10 mMTris-HCl, 1 mM EDTA buffer (pH 7.4) and 5 ml (5×1 ml) water,to remove excess DNAfrom surface [2], and placed under the liquid cell filled with TE buffer (pH 7.4).

Preparation of sample for control experiments: Control samples were prepared by treating APmica with TE buffer and with drug only solution (as relevant to drug-DNA solution), keeping preparation method similar to that for the drug-treated DNA sample.

The equation used for freely joined chain (FJC) model: A modified FJC model has been applied to fit the melting region of the force-extension curves of free DNA and drug-treated DNA molecules [1]:


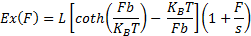


where, *L* is the contour length, *b* is the Kuhn length and *s* is the stretch modulus of single stranded DNA. *K_B_* and *T* being the boltzmann constant and experimental temperature respectively, has been directly put into the equation and *L* has been kept near the experimental value to fit the profiles and to extract *b* and *s* values.

Reference:

[1] Smith, S. B., Cui, Y., Bustamante, C., 1996. Overstretching B-DNA: the elastic response of individual double-stranded and single-stranded DNA molecules. Science 271, 795-799.


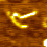

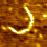

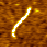

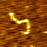

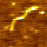

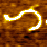


**Figure A.** AFM topographic images of free 692 bp dsDNA fragments are shown at single molecule resolution (DNA concentration: 1.7 µgml^-1^dle bar: s (table 1).e complexes increases gradually with increasing 000000000000000000000000000000000000000000000000000000000). Scale bar: 100 nm, Z-range: 0-0.6 nm.

217.0±25.7 nm

**0 hr**

**1 hr**

239.7±21.3 nm

**6 hr**

247.2±19.3 nm

**12 hr**

253.0±17.3 nm

**18 hr**

250.2±19.8 nm

**36 hr**

270.2±18.8 nm

**48 hr**

273.0±15.3 nm

**Figure B.** Contour length distribution diagrams of dsDNA fragments (with their most probable values) before and after treatment with nogalamycin.

217.0±25.7 nm

**0 hr**

**1 hr**

253.2±16.6 nm

**18 hr**

254.0±17.5 nm

**48 hr**

259.0±14.4 nm

**36 hr**

249.0±17.0 nm

**Figure C.** Contour length distribution diagrams of dsDNA fragments (with their most probable values) before and after treatment with daunomycin.

| Type of sample  (t_incub_) | Most probable contour length (nm) (±SD) | Change in contour length (nm) | % increase in length |
| --- | --- | --- | --- |
| Free DNA | 217.0±25.7 | 0 | 0 |
| DNA-Drug complex  (t_incub_: 1 h) | 239.7±21.3 | 22.0 | 10.1 |
| DNA-Drug complex  (t_incub_: 6 h) | 247.2±19.3 | 30.2 | 13.9 |
| DNA-Drug complex  (t_incub_: 12 h) | 253.0±17.3 | 36.0 | 16.6 |
| DNA-Drug complex  (t_incub_: 18 h) | 250.2±19.8 | 33.2 | 15.3 |
| DNA-Drug complex  (t_incub_: 36 h) | 270.2±18.8 | 53.2 | 24.5 |
| DNA-Drug complex  (t_incub_: 48 h) | 273.0±15.3 | 56.0 | 25.8 |

**Table A.** AFM-derived most probable DNA contour length values, before and after nogalamycin treatment.

| Type of sample  (t_incub_) | Most probable contour length (nm) (±SD) | Change in contour length (nm) | % increase in length |
| --- | --- | --- | --- |
| Free DNA | 217.0±25.7 | 0 | 0 |
| DNA-Drug complex  (t_incub_: 1 h) | 253.2±16.6 | 36.2 | 16.7 |
| DNA-Drug complex  (t_incub_: 18 h) | 254.0±17.5 | 37.0 | 17.1 |
| DNA-Drug complex  (t_incub_: 36 h) | 249.0±17.0 | 32.0 | 14.7 |
| DNA-Drug complex  (t_incub_: 48 h) | 259.0±14.4 | 42.0 | 19.4 |

**Table B.** AFM-derived most probable DNA contour length values, before and after daunomycin treatment.

**Figure D.** Representative AFM force-extension trace for 692 bp dsDNA molecule.

**(E)**

**(B)**

**(A)**

**(C)**

**(D)**

**(F)**

**Figure E.** AFM force-extension traces for nogalamycin-treated dsDNA molecules, for dsDNA:drug molar ratio (1:10) after (A) 1 h and (B) 48 h incubation; for the molar ratio (1:26) after (C) 1 h and (D) 48 h incubation; and for the molar ratio 1:40 after (E) 1 h and (F) 48 h incubation.

**(A)**

**(B)**

**(C)**

**(D)**

**Figure F.** Representative AFM force-extension traces for daunomycin-treated dsDNA molecules, for the dsDNA:drug molar ratio (1:10) after (A) 1 h and (B) 48 h incubation; and for the molar ratio (1:26) after (C) 1 h and (D) 48 h incubation.
